# Collectanea Medica: Consisting of Anecdotes, Facts, Extracts, Illustrations, &c.

**Published:** 1819-10

**Authors:** 


### COLLECTANEA MEDICA:

CONSISTING OF

### ANECDOTES, FACTS, EXTRACTS, ILLUSTRATIONS, &c.

***Relating to the History or the Art of Medicine, and the Auxiliary Sciences.***

Floriferis ut apes in saltibus omnia libant,

Omnia nos itidem depascimur aurea dicta.

### *Observations on the Mode in which the Nerves contribute to Secretion in the Glands.*

[Bordeu, *Rechérches sur les Glandes*, § cvi.]

WE should now discover in what manner the nerves act in effecting the secretions: that they do thus act, we have fully proved. It should be remembered, that we have stated that the fluids flow in increased quantity to a gland actually engaged in secretion. Before we proceed further, let us demonstrate this proposition.

Let us only stop to view what takes place in the parotids during a repast. They separate, as every person knows, a large quantity of saliva: all this cannot be said to have been contained in the gland; since, as we have elsewhere proved, it is extremely tumefied when it contains but a little water. Now it is never so tumefied before a repast; it does not then contain as much saliva as will moisten two or three napkins, which has been done with the saliva flowing during a repast.

The blood flows to the parotid in a quantity proportionate to the saliva evacuated, that is evident: there flows, then, more fluid to the gland during the time of secretion than at any other time; or else the same quantity of fluid is carried to it more frequently, which comes to the same thing; for, the repast being ended, the parotid employs the whole time to the ensuing one, in separating only as much saliva as is necessary to moisten the mouth: Is not this quantity less than that separated during a repast?

It is easy to apply to every other gland what has been said respecting the parotid. This phenomenon is evident; and, to explain correctly the secretions, it is necessary to commence by giving proper reasons for them: we must not, certainly, neglect the mode in which they ordinarily take place.

This direction of the fluids towards a gland, cannot depend on the simple action of the heart, or on the general laws of the circulation, which have their influence during the secretion of the saliva as well as at other times.

It cannot be said that the gland is relaxed during the time of secretion; since, from what we have elsewhere demonstrated, it appears, on the contrary, that it becomes harder and more solid; which supposes a state of constriction rather than of relaxation. Besides, have we not often remarked, that the nerves act more forcibly during the secretion than at any other time?

How then may this phenomenon be explained? It may be said that the glands act like cupping-glasses; that they draw, in a manner, the fluids. This phenomenon is a very important one even in the theory of many diseases, in which transports of fluids, quite independant of the ordinary causes of the circulation, are clearly evident.

It must be discerned, from what we have hitherto said, that we think this phenomenon should be attributed to the action of nerves; and it is thus we believe they act. They cause the blood to come to a gland, by directing and augmenting the circulation in that organ, which (if it may be so expressed) makes, when it is in action, a sort of *distinct body:* it employs more blood than is necessary for it during its repose. But how do the nerves act then?

Demonstration on this point must not be expected; but the fact is not the less true, although we cannot well comprehend the reason of it. The following is what we think may be advanced on this subject.

Anatomy teaches us that the nerves accompany all the arteries, and we have even seen certain large vessels, as those of the abdominal cavity, enveloped in nervous-like membranes; the nerves twine about the arteries as they accompany them; they appear to seize a trunk, and pursue it to its extreme ramifications.

Supposing that the arteries which go to a gland receive an increase of force by the nerves which are attached to their parieties, we may conceive that, if these nerves happen to act a little more powerfully than ordinarily, the fluids will be carried proportionably quicker from the trunk of the vessel towards its ramifications, and the trunk then relaxing and opposing less resistance to the column of blood coming from the artery whence it arises, it will in turn receive more blood; and so in succession. In a word, the systole of the vessels of a gland, whilst it is in action, is perhaps much stronger than when it is relaxed; the diastole is also proportionably strong: this is the reason why the fluids come then in larger quantity; and this depends on the action of the nerves, which, parting from a ganglion as from a centre, act vigorously on the vessels they accompany, and make them beat much more powerfully; we do not say they beat more frequently. All this will be hereafter explained.

It must be acknowledged, that if the action of the nerves here is to cause a contraction of the small veins of a gland, this contraction might augment the secreted matter;* but it must take place to a great extent to be a sufficient cause; and it is besides easy to perceive, that the veins of an organ in action receive more blood than when it is relaxed, as is seen in the muscles, and as we have observed in a man bled in the jugular vein,—who, by moving his jaw, made the blood spring from the vein, and the saliva flow into his mouth. We see also that the jugular veins of a person in a state of salivation are at least as full as those of persons not in a state of salivation: this latter phe-

* Bordeu is elsewhere careful to show, that a variation in the degree of nervous influence will cause a difference in the *quality*, as well as in the *quantity*, of the secretion of a gland; and that this is often rendered more poignant by the agency of some of the passions.—Edit.

nomenon may happen, it is true, from the blood not circulating so freely in the veins, which takes place in those who have what are termed transports of fluids to the head: we know not if the veins have not then a tendency to the ANTIPERISTALTIC motion.* This motion, indeed, which we cannot here examine, could only take place from the action of the nerves.

The muscles which are in the vicinity of glands favour also the transport of fluids to the secretory organs: it is apparent that the muscles receive less blood during their contraction; or they may, if it be so desired, receive more then. These two hypotheses do not regard what we are at present considering; it is enough that it appears, the motions of the muscles occasion that of the fluids. We have elsewhere spoken of this action of the muscles in respect to the glands.

*On the mode in which the choice of the fluids is effected, or the secretion, properly speaking.*—This is the principal question: It is to know why the most subtile of the fluids do not pass into a vessel where the grosser fluids pass; and why that constantly happens, so that the same gland ordinarily separates the same fluid, &c.

The nerves act in a gland; they prepare the organ, and direct the fluids in its vessels; they make the blood, which contains all sorts of fluids, come to it. What, then, is the power which chooses and separates the different parts one from the other?

Are not the nerves themselves the principal cause of this separation? It is thus we may conceive that it is effected: The fluids carried into the vessels, or (if it be required) into the follicles of the glands, have only two courses to take,—that of the secretory vessel or that of the vein, or perhaps that of the venous lymphatics; the fluids, still wholly mingled together, come striking against the orifices of the little veins or of the secretory vessel; but these orifices are each guarded with a sort of little sphincter and some nervous fibrils; they may then contract or dilate according as may be requisite, and this will happen according to the irritation made on the nerves: a too forcible impression will close the secretory orifice; a too feeble one will not irritate it sufficiently to cause it to open. There must be a certain relation between the matter which makes the effort to open the sphincter, and the nerves which direct its orifice.

Secretion is then reduced to a species of *sensation*, if we may so express it: the matters proper to excite a certain sensation will pass, and others will be rejected; each gland, each orifice, will have, if it may be so termed, its particular *taste;* all that is foreign to it will be rejected on ordinary occasions.

The tension that the titillations, and the little irritations proportionate to the tone of the nerve, will procure, will effect the secretion; the sphincter, directed by nerves *attentive*, and insensible to all that

* As the possibility of such a motion of the veins is not, we believe, generally suspected, we may observe that it seems to be proved by an experiment made by BELLINI and HALLER, and repeated by SPALLANZANI, from which it results, that, if an artery be pricked, the blood retrogrades towards the place of the wound, not only from the extremities of the artery, but also from the trunk of the correspondent vein.—EDIT.

does not regard them, will not let any matter pass until it has given good proofs of its propriety; all will be arrested; the good will be taken, and the bad sent away elsewhere.

The nerves of each gland have their particular tone, as well as those of all the other organs. It has been said that we should see with the foot, if the organ and its nerves were properly disposed; we may say also, that the separation of bile would be effected in the mouth, if the nerves of the parotid gland had another sort of sensibility, or, if we may so venture to express it, another *taste.*

---

### COLLECTION OF RARE AND INTERESTING CASES IN PHYSIOLOGY AND PATHOLOGY.

[*Continued from page* 205.]

*Abolition of Sensibility.*—There was at the *Bicêtre*, in 1808, a man, 50 years of age, who, for eighteen years, had the right arm deprived of sensibility. The member did not diminish in bulk; he performed every motion with it with the same agility and the same force as with the healthy arm. A phlegmon, accompanied with heat, redness, and tension, took place in it, without the patient experiencing from it the least pain. This person could plunge his arm into boiling water, without any redness being manifest from it. However, a pot of boiling leys having fallen on the hand, wounds ensued, that were a long time before they were cured. This insensibility came on after a fall on the prominence of the shoulder; where several cicatrixes might still be seen. In 1807, when he was scraping up some mortar with a shovel, he felt something crack in his hand; he thought he had broken his shovel; but, perceiving that his fore-arm was bent, he discontinued his labour, but did not present himself at the infirmary until the following day, not experiencing any pain. The two bones were fractured. The patient felt no uneasiness during the reduction of the fracture, notwithstanding the forcible extension that it was necessary to employ.—*Case published by* M. Hébréard, *second Surgeon of the Bicêtre.*

*Paralysis.*—Wepfer relates, that an hemiplegic old man presented the singular phenomenon of a jaundice affecting only the diseased side. The half of the body was so exactly tinctured, that the nose was yellow on one side, whilst the other half of the same part of the face was of the natural colour.

*Metastasis.*—Cruikshanks witnessed a spitting of pus in a man who had a fistula in ano. This spitting ceased as soon as the fistula was cured by the operation.

Assalini relates, that a woman after delivery was attacked with a fistulous ulcer in the middle of the thigh. During nine years this ulcer furnished a matter similar to milk. Assalini states that he has seen two other women, who evacuated milk by the navel.

Dr. Gastellier, in his treatise on the acute diseases of puerperal women, relates the following case: A lady of Nemours sent for Dr. Gastellier, to consult him respecting a collection of matter in the inferior part of the left leg, near the malleolus externus. This collection, occurring subsequent to phlegmonous erysipelas, had externally all the signs of gangrene: it presented a very considerable surface, and fluctuation in it was very evident. M. Gastellier proposed to make an opening into it; but it was late in the evening, and the patient had then much repugnance to the operation: however, this physician persuaded her to consent to have it done on the ensuing morning. On this day M. Gastellier found his patient in a state of great uneasiness; she was suffering cardialgia and vomitings of purulent matter. On examining the diseased part, he could perceive neither swelling nor fluctuation, nor any appearance of a collection; all had disappeared. An emetic was administered, and the patient vomited a prodigious quantity of white and well-formed pus. Being purged the following day, she voided by stool a large quantity of similar matter: this was repeated five or six times, each dose producing the evacuation of a certain quantity of purulent matter. The cardialgia only subsided under the use of purgatives. The idea of an operation she dreaded, caused in this patient a revolution, which determined this metastasis.

Lemery relates the case of a priest, who suffered for eight years a periodical vomiting, the circumstances of which were very rare. Five hours before he vomited, the patient experienced very violent pain about the loins; the vomiting continued during four or five hours, with some intervals. The matter vomited was of a deep-red colour; this was only water having a strong urinous odour. The patient ate but little; drank only wine, but of this copiously. As soon as the vomiting ceased, he felt very well. Exercise was salutary to him; he suffered much more when he neglected to resort to it.*

---

*Observations on some Electrical Phenomena of different Substances placed on Water.* By M. Virey.

Some remarks have already been made on this subject, and the gyratory motions of little fragments of camphor on water have been attributed to the attraction of the liquid to effect their solution; but we shall show that they appear rather to result from electricity.

1°. *The phenomena of rotation.*—Having placed on pure water, at the temperature of about 12° centigrade (about 60° Fahrenheit), different fragments of camphor, we saw them first spontaneously turn on their own axis by a revolutory motion, which sensibly accelerated until it formed a sort of vortex. The little fragments, especially, executed this revolution with a very surprising rapidity. Soon afterwards we often saw these fragments describe an orbit, in the manner of the planets, which roll in celestial space in the double motion of rotation and circulation. When one of the larger molecules turns near one much smaller, the latter ordinarily ceases to turn; for it seems that its motive force is absorbed: just as the moon, in the terrestrial orbit, only turns very slowly on its own axis. The little fragments only commence their motion when the larger molecule has

* See the xxxth and xxxist volumes of this Journal, for some interesting discussion on this subject; especially in the observations of Dr. Yeats.

passed to some distance from them. We have remarked that the lesser molecules often make their rotation from right to left, whilst the greater fragments have appeared to us to effect theirs from left to right. It is rare that the fragments turn in a different direction from that which they first began to take.

2°. *Of the phenomena of attraction and repulsion.*—Most frequently the fragments attract each other: it is towards a projecting angle of a larger molecule that the lesser ones are ordinarily drawn. Each fragment has its poles, and they are differently electrified; for, if there are some which attract, there are others which evidently repel, each other: it proves that there are poles of those different kinds. Fragments of common resin, and other resinous substances, equally exert mutual attractions. Shavings of dry wood, more or less resinous, also attract each other in a similar manner. The gums, on the contrary, always mutually repel each other; we see them endeavour to separate. This effect is more evident in the gum tragacanth in powder, than in gum arabic, or common gum of the trees of our own country. The latter are much less readily soluble than the tragacanth.

3°. *Of the influence of conductors of electricity.*—When we approach a rod of iron, or of other metal, very near the fragments of camphor forming vortices on the water, but without touching them, we see these fragments immediately arrested in a remarkable manner. It must be supposed that these metallic conductors should be at a very short distance from the fragment, since the electricity is very weak, otherwise the production of this phenomenon is not obtained. We have not observed that the metallic conductors oppose the attractions of fragments of resin on water; neither do they oppose the repulsions of pieces of gum. It appears, then, that electricity performs a part in many of these phenomena, although it may be difficult to determine its exact degree. The revolution of camphor cannot be the effect of its dissolution, as it has been stated, since the hydro-chlorate (muriate) of ammonia, and other soluble substances, turn but very feebly on their axis; yet they dissolve in water much better than camphor. —*Journal de Pharmacie,* Mai 1819.

---

### *An Alphabetical List of the Plants and other Articles of the Materia Medica mentioned in the Works of* Hippocrates, *with the Latin Version of* Föes, *and the Synonyms of* Linnæus. [Extracted from a Memoir by Dr. Paulet.]

Αβροτονον, Hippoc.; abrotanum, Föes; artemisia abrotanum, Linn.

Ακανθα et *ακανθα αιγυπτια*; spina et spina ægyptia; mimosa nilotica.

Ακανθα λευκη; spina alba; euphorbia nerifolia?

Αχρας; pyrus sylvestris; pyrus communis.

Ακτη; sambucus; sambucus nigra.

Ακυλος et *ακυλον*; glans ilicis; *fruit du* quercus faginea, Lamarck.

Αδιαντον; adiantum; asplenium ceterac.

Αδιαντον *καλλιφυλλον*; adiantum; adiantum capillus Veneris.

Αγνος; vitex et agnus castus; vitex agnus castus.

Αγνος λευκη; agnus albus; vitex leucoxylon.

Αιγειρος; populus nigra; populus nigra.

Αιγειρος κρητικη; populus cretica; populus græca, Ayton.

Αιγος κέρας; fœnum græcum; trigonella fœnum græcum.

Αιρα; lolium; holcus lanatus.

Αλφιτον; polenta; *fruits de l'*hordeum sativum *mondés et en cataplasme par leur décoction dans l'eau et le vin.*

Αμαμελις; amamelis; *fruit du* mespilus germanica.

Αμαρακος; amaracus; origanum marjorana.

Αμωμον; amomum; amomum racemosum, Lamarck.

Αμπελος; vitis; vitis vinifera.

Αμπελος αγρια; vitis sylvestris; vitis sylvestris, Tournefort?

Αμυγδαλη; amygdala; *fruits de l'*amygdalus communis.

Αναγαλλις; anagallis; anagallis arvensis.

Αγχουσα; anchusa; anchusa officinalis.

Ανδραχνη; portulaca; portulaca oleracea.

Ανδραχνη αγρια; portulaca sylvestris; euphorbia peplis.

Ανδραχνη ποταμιη; portulaea fluviatilis; peplis portula.

Ανδραφαξις; atriplex; atriplex hortensis.

Ανδραφαξις αγρια; atriplex agrestis; chenopodium bonus Henricus.

Ανεμονη; anemone; anemone pulsatilla.

Ανεθον; anethum et mentha; anethum graveolens.

Ανισον; anisum; pimpinella anisum.

Ανθεμον; anthemum; anthemis nobilis.

Απαρινη; aparine; galium aparine?

Απιος et *απιον*; pyrum, pyra; pyrus communis, *et son fruit.*

Αψινθιον; absinthium; artemisia absinthium.

Αρκευθιδες, *αρκευθου καρπον*; juniperi baccæ; *baies du* juniperus communis.

Αρκευθος; juniperus; juniperis communis.

Αριστολοχια; aristolochia; aristolochia rotunda.

Αρον; arum; arum maculatum.

Αρον μεγα; dracunculus magnus; arum dracunculus.

Αρτεμισιη; artemisia; artemisia vulgaris.

Ασπαλαθος; aspalathus; amyris kafal, Forskael?

Ασπαραγος; asparagus; asparagus officinalis.

Ασφοδελος; asphodelus; asphodelus luteus et A. ramosus.

Ασταφις; uva passa: *raisins secs.*

Βακχαρις; Baccar et Baccharis; salvia sclarea.

Βαλανος; quercûs glans; *fruit du* quercus esculus.

Βαλανον αιγυπτιον; glans ægyptiaca; *fruit du* guilondina moringa.

Βατος; rubus; rubus fruticosus.

Βατραχιον; ranunculus; ranunculus aquatilis.

Βηχιον; bechium; tussilago farfara.

Βληχον; pulegium; *voyez γληχον.*

Βλιτον ou βλιτον; blitum; amaranthus blitum.

Βολβιδιον; bulbulus; allium ascalonicum.

Βολβιον; bolbium et bulbulus; œnanthe pimpinelloïdes.

Βολβιτον; bolbitam; hyacinthus comosus.

Βολβος; bulbus; allium altaïcum, Pallas.

Βολβος λευκος; bulbus albus; ornithogalum umbellatum.

Βοτανη; herba; cyromoriom coccineum?

Βοτρυς, *βοτρυες*; uvarum racemi; *grappes de raisin.*

Βραθυς; sabina; juniperus sabina.

Βρομος; avena; avena alatior.

Βρυον; muscus; lichen albidus, Schrank.

Βρυον θαλασσιον; mucus marinus, fucus, alga marina; uva lactuca.

Βρυονιη; bryonia; bryonia alba.

Βυκερας ou *βουκερας*; fœnum græcum; trigonella fœnum græcum.

[*To be continued.*]

